# Towards Accurate Breslow Measurements: Mitigating Issues in Histopathological Imaging

**DOI:** 10.3390/e28060643

**Published:** 2026-06-08

**Authors:** Nico Curti, Lorenzo Dall’Olio, Giulia Veronesi, Giulia Querzoli, Federico Venturi, Azzurra Sisi, Sara Peluso, Gastone Castellani, Emi Dika

**Affiliations:** 1 Department of Physics and Astronomy, University of Bologna, 40127 Bologna, Italy; nico.curti2@unibo.it; 2 Data Science and Bioinformatics Laboratory, IRCCS Istituto delle Scienze Neurologiche di Bologna, 40139 Bologna, Italy; lorenzo.dallolio@ausl.bologna.it; 3 Department of Medical and Surgical Science, University of Bologna, 40138 Bologna, Italy; giulia.veronesi.md@gmail.com (G.V.); azzurra.sisi2@unibo.it (A.S.); sara.peluso5@unibo.it (S.P.); gastone.castellani@unibo.it (G.C.); emi.dika3@unibo.it (E.D.); 4 Oncologic Dermatology Unit, IRCCS Azienda Ospedaliero-Universitaria di Bologna, 40138 Bologna, Italy; 5 Pathology Unit, IRCCS Azienda Ospedaliero-Universitaria di Bologna, 40138 Bologna, Italy; querzoligiulia@gmail.com; 6 IRCCS Azienda Ospedaliero-Universitaria di Bologna, 40138 Bologna, Italy

**Keywords:** melanoma, Breslow, artificial intelligence, digital pathology, computer vision

## Abstract

Breslow thickness is a key prognostic parameter in the staging of cutaneous melanoma, but its manual measurement is affected by operator dependency and the complex morphology of the epidermis. Identifying both the deepest melanocyte and the correct perpendicular path to the epidermal surface can be challenging, especially in highly irregular tissues. This study investigates a more robust estimation of Breslow thickness through the development of a semi-automated Computer Vision–based software. Inter-operator variability was assessed by comparing measurements performed by seven histopathologists on 40 Whole Slide Images of non-ulcerated pT1a melanomas with Breslow thickness below 0.8 mm. The agreement between human measurements and AI results was evaluated. Significant variability in the orientation of the measurement segments was observed, highlighting the difficulty for human operators in identifying the correct perpendicular direction. A linear relationship was found between angular variance and variance in Breslow thickness values, linking epidermal irregularity to increased measurement uncertainty. Overall, statistically significant differences were observed between the AI system and five of the seven operators, indicating a general tendency among experts to overestimate Breslow thickness.

## 1. Introduction

The incidence of cutaneous melanoma has increased in recent decades, likely due to a complex interplay of environmental and genetic factors [1,2,3,4]. Epidemiological projections reinforce this trend on a global scale: approximately 331,700 new cases and 58,700 deaths attributable to cutaneous melanoma were recorded worldwide in 2022 alone, and the absolute burden is expected to continue rising substantially over the coming decades [5]. This growing public health challenge underscores the critical importance of reliable and reproducible histopathological assessment of primary tumors, as diagnostic precision at the time of initial staging directly determines treatment strategy, follow-up intensity, and long-term patient outcomes. In this context, an accurate assessment of progression is crucial, with clinical and pathological predictors emerging as important tools for estimating survival [1,6,7,8,9]. The current staging system for melanoma is based on the 8th edition of the TNM classification established by the American Joint Committee on Cancer (AJCC) [10,11]. Among the most clinically consequential changes introduced in the AJCC 8th edition was the redefinition of the T1 category, which introduced the 0.8 mm Breslow thickness threshold as the boundary separating T1a from T1b melanomas. According to this framework, T1a is defined as a non-ulcerated melanoma with Breslow thickness strictly below 0.8 mm, while T1b encompasses either a non-ulcerated lesion measuring 0.8–1.0 mm or an ulcerated lesion below 0.8 mm [12]. This threshold has direct implications for sentinel lymph node biopsy (SLNB) recommendations, excision margin planning, and follow-up scheduling, making measurement precision around this cut-off especially consequential in daily pathological practice. This staging system influences critical decisions such as treatment selection and follow-up strategies.

In the TNM classification, Breslow thickness (pT category) is considered the most important histopathological predictor of metastasis risk and patient survival [12,13,14]. Contemporary reviews confirm that Breslow thickness remains the single most important predictor of outcome in primary melanoma, ahead of patient age and ulceration [15]. Beyond its role in staging, Breslow thickness directly informs the recommended surgical excision margin: invasive melanomas below 1 mm are generally managed with a 10 mm radial margin, those between 1 and 2 mm with 10–20 mm, and lesions exceeding 2 mm with a 20 mm margin [15]. Even small systematic measurement errors may therefore indirectly affect the extent of surgical intervention recommended to the patient. Imprecision in the measurement of Breslow thickness, therefore, has the potential to significantly impact the clinical evaluation of patients, affecting prognostic estimates and decision management [13,16,17,18,19,20].

The standard clinical practice for Breslow thickness assessment is based on microscopic examination of formalin-fixed, paraffin-embedded tissue sections stained with hematoxylin and eosin (H&E). From a pre-analytical standpoint, biopsy technique is a critical determinant of measurement reliability. The preferred approach for a clinically suspicious melanocytic lesion is a complete excisional biopsy with narrow margins, typically 1–3 mm, as this method ensures that the entire apparent lesion is removed intact and provides the pathologist with the maximum opportunity to assess the full depth of invasion [15,16]. Superficial shave biopsies are generally discouraged because they may transect the deep margin of the lesion, prevent accurate determination of the full Breslow thickness, and potentially lead to underestimation of tumor depth [16]. In anatomically challenging sites such as the face, palms, soles, or digits, full-thickness incisional or punch biopsy of the clinically thickest or most atypical portion of the lesion may be acceptable, provided that the chosen area is representative of the maximum invasive depth [16]. When a partial biopsy is unavoidable, and the base of the lesion is transected, the pathologist should report the thickness as “at least” the measured value, noting that the true Breslow thickness cannot be definitively established from that specimen alone, and correlation with the re-excision specimen is required [21]. Optimal histological evaluation requires well-cut, well-stained H&E sections prepared from formalin-fixed paraffin-embedded tissue; frozen sections are strongly discouraged due to the tissue distortion and measurement inaccuracy they introduce [21].

First introduced in 1970, the Breslow measurement gauges the depth of tumor invasion, from the top of the granular layer or, if the epidermis is missing, from the base of the ulcer to the deepest point of the tumor. In clinical practice, this measurement is typically taken manually, using an ocular micrometer calibrated to a microscope [1,21,22,23,24,25]. In non-ulcerated lesions, the measurement originates from the top of the granular layer of the overlying epidermis; in ulcerated melanomas, the reference is the base of the ulcer. According to current reporting recommendations, Breslow thickness should be recorded to the nearest 0.1 mm, with values ending in decimal places 1–4 rounded down and those ending in 5–9 rounded up; for tumors ≤ 1 mm, recording to the nearest 0.01 mm may be practically feasible and is encouraged when it could influence clinical decision-making near the staging thresholds [21]. A critical geometric requirement for measurement accuracy is that tissue sections must be cut as perpendicular as possible to the epidermal surface: the Breslow thickness can only be evaluated accurately in sections cut perpendicular to the skin surface, and any deviation from this orientation may overestimate the true vertical depth of invasion [24]. This geometric challenge is particularly relevant in thin melanomas, where a deviation of just a few degrees from the perpendicular may translate the measured value across clinically important staging thresholds. Furthermore, periadnexal extension, microsatellites, and foci of neurotropism or lymphovascular invasion should not be included in tumor thickness measurements, and in cases where regression is present, the thickness of the regression zone should be noted separately rather than substituted for the Breslow thickness of the invasive component [21].

However, the manual evaluation of Breslow thickness can lead to inter-observer variation and estimation errors [26,27,28,29]. Although Breslow thickness is generally considered more reproducible between pathologists than other melanoma parameters, such as Clark level, mitotic rate, or regression, clinically meaningful discordance can still occur, particularly near staging cut-off values [30]. A recent study on interobserver variability in melanoma histopathological evaluation found a mean level of agreement across all study variables of only moderate strength (Cohen’s kappa = 0.5), and emphasized that even nominally quantitative variables like Breslow thickness are not immune to subjective influences when measurement is performed near the epidermal reference point or when tissue architecture is irregular [30]. Moreover, a large-scale study assessing pathologist variability during the development of the AJCC 8th edition staging system showed that, while average participant Breslow thickness assessments closely approached consensus values, a notable terminal digit preference was observed, with large spikes in reported values ending in 0 or 5, reflecting a rounding bias that may artificially cluster measurements around clinically critical thresholds [31]. A recent landmark study assessing the discordance, accuracy, and reproducibility of melanoma diagnosis among eight expert pathologists reviewing 792 melanoma-suspicious lesions found complete agreement in only 53.5% of cases, with considerable discordance, particularly for early-stage and non-invasive lesions, reinforcing the need for computational decision support tools in melanoma pathology [32]. The specific sources of variability in Breslow measurement include differences in identifying the correct epidermal reference point, locating the deepest invasive melanoma cell, interpreting ambiguous dermal cells, managing tangential or oblique sections, and selecting the precise spatial orientation of the measurement segment, the latter being one of the primary foci of the present study. In this context, the introduction of digital pathology, in combination with Artificial Intelligence (AI) models, could play an important role in reducing human subjectivity [33,34,35]. The integration of whole slide imaging (WSI) and AI in digital pathology is rapidly transforming the field by improving analysis accuracy, reproducibility, and throughput.

Recent developments in artificial intelligence and computational modeling across multiple biomedical and data-intensive domains further support the growing role of advanced machine learning approaches in medical image analysis and decision support systems. In particular, adaptive optimization frameworks such as the tri-population evolutionary strategy proposed by Chen et al. for constrained multi-objective optimization problems highlight the importance of dynamically balancing robustness, convergence, and uncertainty management in complex computational pipelines [36]. Similarly, attention-based spatio-temporal graph neural networks, such as the AST-GNN architecture introduced by Zhou et al., have demonstrated the ability of graph-based deep learning models to capture complex spatial relationships and contextual dependencies in highly heterogeneous data environments [37]. In parallel, heterogeneous multi-view representation learning approaches have shown promise in integrating diverse sources of information into unified computational models, as demonstrated by Zhao and Yu in AI-enabled learner modeling systems [38]. Collectively, these methodological advances reinforce the broader trend toward explainable, geometry-aware, and context-sensitive AI systems, which are increasingly relevant for computational pathology applications requiring reproducible quantitative measurements from complex histological structures.

Deep learning has shown promising outcomes in diminishing subjective interpretation in dermatopathology, with systematic reviews identifying a broad body of evidence supporting the use of these methods in melanoma histology for tasks, including melanocytic lesion classification, segmentation of tumor regions in WSIs, mitosis detection, and prognostic feature extraction [39,40]. A recent narrative review of digital pathology and AI in melanoma diagnostics categorized current research across five domains: WSI-based classification, histological feature extraction, spatial immune profiling, molecular marker prediction, and explainable AI approaches, collectively illustrating the breadth of ongoing efforts to automate and standardize pathological assessment [41]. Regarding Breslow thickness specifically, a recent narrative review of emerging AI models for dermoscopy-based preoperative estimation of tumor depth found that, while deep learning approaches are under active investigation, current models still exhibit substantially lower accuracy for thin melanomas (≤1.0 mm), which is precisely the subgroup with the greatest staging boundary sensitivity [42]. The problem of AI-assisted histopathological Breslow measurement on WSIs, rather than preoperative dermoscopic prediction, has received comparatively little attention, and no validated, reproducible, geometry-aware tool has yet been established for routine use.

Despite the advances described above, several structural limitations of existing measurement approaches—both manual and computational—remain inadequately addressed in the current literature and directly motivate the present work. A first fundamental limitation derives from the maximum-based definition of Breslow thickness itself: because the measurement is anchored to the single deepest invasive cell observed across the entire tumor cross-section, it is inherently sensitive to outlier findings. A single ambiguous or atypical deeply positioned cell—difficult to confirm unambiguously as truly invasive rather than displaced, entrapped, or periadnexal—is sufficient to drive the reported value upward, potentially shifting the tumor across a staging boundary [43,44]. This outlier sensitivity is intrinsic to the maximum-based definition and cannot be resolved by improved staining or higher magnification alone; it requires explicit identification and contextual evaluation of candidate deepest cells, a task that benefits from computational support. A second and closely related limitation concerns the correct identification of the measurement orientation. Even when the deepest cell is correctly identified, the measurement must be drawn perpendicularly to the local epidermal surface at the point of reference. On a curved, undulating, or irregularly folded epidermal contour—as is commonly encountered in formalin-fixed tissue sections—the local perpendicular direction changes continuously along the surface and cannot be reliably inferred by visual inspection alone, even on a high-resolution digital WSI [24]. Operators working with digital measurement tools on WSI platforms have access to calibrated on-screen rulers, which solve the problem of scale but leave angle selection entirely to the user. As a result, different operators drawing measurement segments by eye will naturally disagree on the angle of the segment relative to the epidermal surface, and any deviation from the true local perpendicular produces an oblique segment that geometrically overestimates the true vertical depth of invasion. This operator-dependent angular misalignment is a systematic rather than random source of error, and its contribution to inter-observer variability in Breslow thickness has not been quantitatively characterized in the existing literature. A third limitation of current AI-based solutions is that deep learning models developed for histopathological WSI analysis of melanoma have predominantly been trained on small, often single-center datasets—with dataset sizes in the published literature ranging from fewer than 10 to approximately 18,000 images and a median of approximately 324 images—and have relied on cross-validation or split-sample testing rather than independent prospective external validation [39]. Crucially, none of the AI tools reported to date explicitly models or corrects for the angular alignment of the Breslow measurement segment, nor quantifies the relationship between epidermal surface irregularity and measurement uncertainty. This gap leaves pathologists without a geometry-aware decision-support tool for the specific problem of reproducible orientation-corrected Breslow thickness estimation on WSIs of thin melanomas near the critical 0.8 mm staging threshold.

In this study, we aim to analyze the extent of inter-operator variability and imprecision in the measurement of Breslow thickness. To this end, we developed and implemented semi-automated software based on an AI pipeline, which we also used as a benchmark for the assessment of the manual measurements performed by different operators. Leveraging the results generated by the AI model, a valid alternative for the estimation of Breslow thickness is also proposed. The study specifically focuses on non-ulcerated pT1a melanomas with Breslow thickness below 0.8 mm, a subgroup in which small absolute measurement differences may alter pT classification and influence downstream clinical decisions, and where the geometric challenges of defining a perpendicular measurement path through an irregular epidermis are most consequential.

## 2. Materials and Methods

### 2.1. The Dataset

This retrospective study was conducted at the IRCCS Sant’Orsola-Malpighi Hospital of the University of Bologna (Bologna, Italy), following approval by the local medical ethics committee, as per the Declaration of Helsinki. The dataset consisted of digitized histological Hematoxylin and Eosin (H&E) stained Whole Slide Images (WSI) of 40 patients affected by melanoma, which were collected between March 2020 and April 2021 by means of deep excisional biopsies or wide excisions. Digitization of the WSI samples was performed using a Nano-Zoomer 2.0-RS Hamamatsu scanner (Hamamatsu Photonics K.K., Hamamatsu City, Japan) with a 40× (0.23 μm/pixel) magnification and autofocusing. The dataset considered in this study was already introduced and used in a previous work [44].

The WSI slides were then reviewed independently by two expert dermatopathologists, both with more than five years of experience in dermato-oncology. Histological evaluations included a detailed description of the tissue and estimation of Breslow thickness. The latter was performed by an expert dermatopathologist (henceforth referred to as Operator 1), making use of a micrometer calibrated to a laboratory microscope, as is standard clinical practice.

Due to the nature of the measuring procedure, no reference points were collected during the assessment, and only the Breslow thickness values were recorded. The results were reported with a precision of centi-millimeter (according to standard clinical practice) and used as a reference for the statistical analyses that follow. A Breslow thickness value ≤ 0.8 mm was obtained for all the analyzed samples, which were therefore classified as pT1a, where the ‘a’ indicates the absence of ulceration.

### 2.2. Inter-Operator Analysis

Given the lack of annotations in the standard manual estimation of Breslow thickness, as performed by Operator 1, we asked a second expert histopathologist (henceforth referred to as Operator 2) to repeat the Breslow thickness measurement in a blind evaluation of the entire dataset. The measurement was this time performed on the digitized WSI slides, using the Sedeen Viewer software (v5.4.4) supported by the Pathology Image Informatics Platform (PIIP) [45].

Operator 2 was asked to draw a segment between the deepest melanocyte that could be manually identified in the slice and the granular layer surface. For each segment, the coordinates of the starting and ending points were saved at the pixel level and converted to micrometers according to the scale factor of the WSI sample. No time limit was set for the measurement, making it possible for Operator 2 to use the full magnification range (between 0.1× and 40×) as needed. Breslow thickness was finally computed as the Euclidean distance between the starting and ending points.

The accordance between the Breslow thickness values obtained by Operator 1 and Operator 2 was then assessed. Since the Breslow thickness values measured by Operators 1 and 2 were obtained from the same samples, and are therefore dependent, a paired *t*-test was performed, considering, for each of the 40 samples, the pair of Breslow measurements obtained by the two operators.

Next, seeing that the estimation of Breslow thickness is also affected by the choice of the starting and ending points of the segment, we asked five additional histopathologists (Operators 3–7) to repeat the measurements over the entire WSI set. This way, not only was it possible to monitor the variability in the Breslow thickness values, but also in the orientation of the segments drawn by the operators. The orientation of the segments was measured in terms of the slope of the line passing through the starting and ending points. Operators 3–7 used as the starting point of the coordinates of the deepest melanocyte identified by Operator 2.

A paired *t*-test between the Breslow thickness measurements obtained by Operators 1–7 was then performed pairwise, assessing the agreement amongst each pair of operators, for each sample of the dataset. Considering once again the lack of annotations in the measurements performed by Operator 1, the orientation variability was assessed on the measurements carried out by Operators 2–7. This was performed by considering the standard deviation of the distribution of the segments’ angles. Moreover, the variability in the angles was tested against the variability of the Breslow thickness values via linear regression. This made it possible to investigate how much of the Breslow thickness variance originated from the choice of the ending point on the granular layer surface.

### 2.3. Semi-Automated Evaluation of the Breslow Thickness

**Figure 1:**
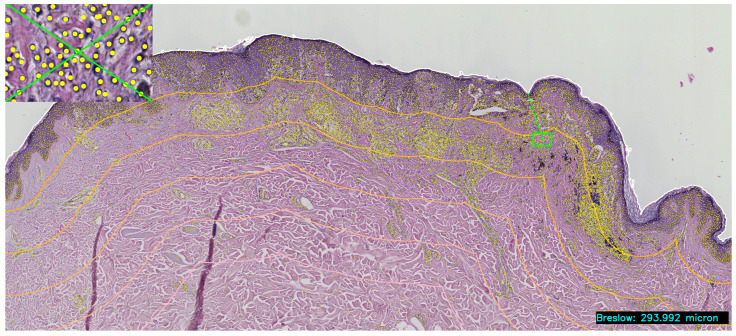
**Breslow semi-automatic estimation.** User interface of the semi-automated software developed for the Breslow evaluation on a WSI sample example. The image shows an intermediate step (user interface) of the developed pipeline applied to an identified ROI of the entire WSI sample. AI models are employed to identify all cells (yellow dots) in the tissue and delineate the granular layer surface of the tissue (white line). The coordinates of each identified cell are used to automatically compute the geodesic distance (green line) from the granular layer surface, which is then displayed on the lower-right corner of the viewer. In the upper-left corner, a high-resolution region of interest (ROI) at 40× magnification is shown to ensure the accurate identification of cell populations. Depth isolines from the granular layer are displayed in varying intensities of orange. Users can manually select the desired location for the measurement, obtaining the Breslow thickness at that point with higher precision than in standard clinical practice.

Dedicated computer vision software was developed for the semi-automated analysis of the digitized WSI samples (ref. Figure 1).

An initial rectangular region of interest (ROI) close to the epidermis surface is manually selected by the user. The ROI must include a sufficiently large portion of the epidermis surface, as well as the complete number of cells to be considered for the measurement. Cells located near the slice borders, which could potentially lead to misleading measurements, should instead be omitted.

The background component is easily filtered out using the Otsu thresholding algorithm [46], and the granular layer is identified as the contour of the resulting binary mask. Next, using a pre-trained U-Net neural network model (EfficientNet-b3 backbone, trained using the standard PanNuke dataset [47] with IoU score achieved on 20% of the images used as a validation set of 0.81 ± 0.09), the entire set of cells is segmented: using 40× magnification overlapping patches, the model produced a binary segmentation of the entire set of cells in the ROI. Feature extraction is then performed to obtain the centroids of the cells from their respective shape contours. The centroid of each segmented cell is used as a marker for the cell’s position in the tissue. The detailed description of the developed AI-based pipeline goes beyond the scope of the current manuscript and its effectiveness related to melanoma applications and robustness in terms of scanner magnification/factory has already been discussed in previous work [44]. The public link to the code developed for this step of processing is available in the Data Availability Statement. We would like to remark that the use of an automated segmentation model for cell recognition represents only a proxy for the analysis of the entire cell population, and it can be easily changed or improved with other kinds of approaches.

Finally, using the coordinates of the cells’ centroids, the minimum distance, i.e., the geodesic distance, from the tissue surface is computed, representing the Breslow thickness. This procedure is repeated for each cell in the set. This way, a set of geodesic distances (according to the Euclidean metric) is obtained, describing the spatial distribution of cells. The possibility to estimate the distribution of geodesic distances provides a robust and automated solution for the correct estimation of the Breslow distance, leaving the possibility of a manually curated measurement (selecting the desired cell by hand) and/or monitoring the global spatial distribution of this metric, which is unpracticable by any human (and expert clinician).

We would like to point out that the development of a fully automated calculation of Breslow thickness would require the classification of all segmented cells. Although several AI solutions were proposed to address this kind of task [48,49,50], their integration requires validation, which is beyond the scope of this study. The release of the semi-automated software will be discussed in future work by the authors.

The Breslow thickness values outputted by the semi-automated software were then compared with the results obtained by Operators 1–7 via a paired *t*-test.

## 3. Results

### 3.1. Inter-Operator Analysis

In the first part of the inter-operator analysis, we evaluated the agreement between the Breslow thickness measurements performed by Operator 1 and Operator 2. We would like to stress that, since Operator 1 conducted measurements without the use of digitized data, no information on the starting and ending points used to determine the Breslow thickness was available. For this reason, the two sets of measurements could only be compared in terms of the Breslow thickness value, without considering the effect of the geometry of the measurement. The paired *t*-test between the two sets of measurements over the 40 available samples produced a *p*-value of 0.47, showing no significant disagreement between the two operators.

**Figure 2:**
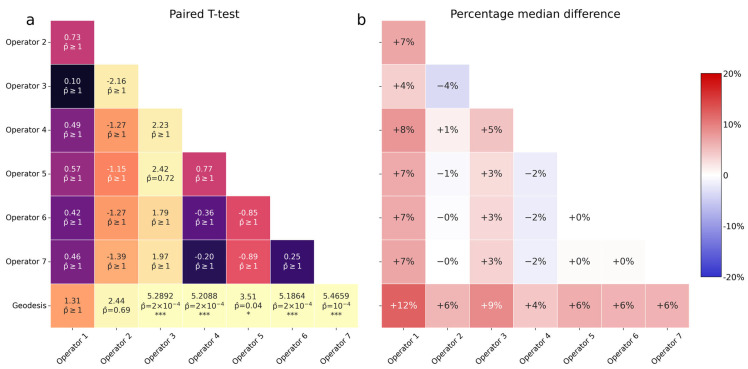
**Inter-operator agreement.** Statistical results obtained from the comparison of the Breslow measurements collected by 7 histopathologists and the geodesic distance. (**a**) Paired *t*-test between the 8 sets of Breslow measurements: for each test, we reported the T statistic value, the corresponding *p*-value, and the adjusted *p*-value corrected by multiple tests (Benjamini–Hochberg); the statistical significance of the test was evaluated on the adjusted *p*-value, using one or more stars (*) for the nomenclature of the levels (0.05, 0.01, 0.001). (**b**) Percentage of difference between the median values of the 8 sets of Breslow measurements: for each combination of variables, we evaluated the difference between the two medians, normalizing the results by the first value; red indicates a positive percentage difference, whereas negative values are displayed in blue.

**Figure 3:**
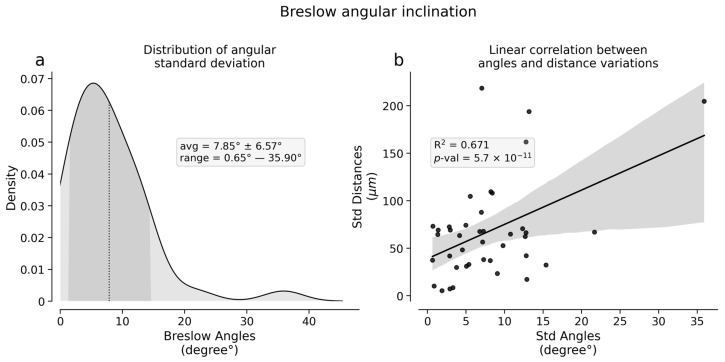
**Human estimated Breslow angular inclination.** Statistical results obtained from the comparison of the angular inclinations produced by 6 histopathologists when performing Breslow thickness measurements, starting from the same melanocyte. (**a**) Distribution of the 40 standard deviations (1 for each image). Each standard deviation is computed among the angles for the shortest path to skin identified by 6 histopathologists over the same digital image; the average angle values ± their standard error and the range of the distribution domain are reported in the box. (**b**) Linear correlation between the dispersion (estimated through the standard deviation) of the angle values and variability (estimated through the standard deviation) of the resulting Breslow distances; the R^2^ score and corresponding *p*-value of the linear regression are reported in the box. Please notice how the usage of standard deviations is a proxy for a dispersion index, implying that higher dispersion (i.e., more difficulty in identifying the correct shortest path) of the angles correlates with a higher dispersion (i.e., more variability/uncertainty) of the consequent Breslow score.

The second part of the analysis was instead focused on the agreement between the Breslow thickness measurements obtained by Operators 1–7. The results obtained from the paired *t*-test are reported in Figure 2a and demonstrate an overall agreement between the seven operators. The results obtained from the assessment of the variability in the orientation of the segments drawn by Operators 2–7 can be seen in Figure 3. It was found that the angles ranged from a minimum of 0.65° to a maximum of 35.90° (ref. Figure 3a), showing notable variability during the measurements. Moreover, significant linearity (y = 0.32x, R^2^ of 0.67, PCC of 0.82, *p*-value ≤ 0.001) between the variability of the angles and the variability of the Breslow thickness values was found (ref. Figure 3b).

Please notice how the usage of standard deviations within Figure 3 is a proxy for dispersion indexes, therefore showing that a higher dispersion among angles (i.e., more difficulty in identifying the shortest path by histopathologists) results in a higher dispersion (i.e., more uncertainty) in the resulting Breslow score. This result confirms that the variability in the measurement of a distance is, as expected, linked to the variations in the geometry of the acquisition.

### 3.2. Semi-Automated Evaluation of the Breslow Thickness

In this part of the analysis, the Breslow thickness measurements obtained by Operators 1–7 were compared with the Breslow thickness computed as the geodesic distance by the semi-automated software.

The linear regression between the results obtained by Operator 2 and the semi-automated software yielded an R^2^ of 0.99 (y = 0.97x, *p*-value ≤ 0.001), confirming a robust proportionality between the two sets. However, using the semi-automated approach always resulted in a reduction in the estimated Breslow thickness score, proving that human operators always chose not the shortest path to the skin. As a matter of fact, the paired *t*-test between the measurements of Operator 2 and the outputs of the semi-automated software led to a moderately significant *p*-value of 0.02 (ref. Figure 2a). It is clear from this outcome that manually estimating the geodesic distance between a point and a curved line as complex as the granular layer after fixation is particularly challenging, presenting high variability in intra-operator measurements and a small overestimate with respect to the semi-automated geodesic identification.

Figure 2a also points to a statistically significant difference between five out of seven operators and the mathematical geodesic distance. Moreover, by looking at the percentage difference between the medians of the Breslow thickness distributions (ref. Figure 2b), we can notice an overestimation trend from the operators’ part when compared with the semi-automatically computed geodesic distance.

**Figure 4:**
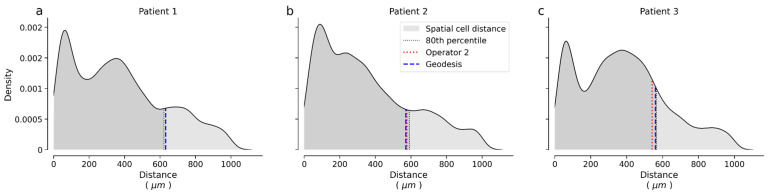
**Spatial Cell Distribution—the 80th percentile suggestion.** Distribution of the distances evaluated from each cell and the corresponding granular layer in 3 different slices. (**a**–**c**) For each cell in the tissue automatically identified by the AI model, we reported the distribution of Euclidean distances between their centroids and the automatically identified granular layer. The manually annotated Breslow distance could be approximated by the 80th percentile of the distribution, or alternatively, by a fixed density value of the cell population, i.e., the distance at which the sparsity of the cells in the tissue surpasses a predetermined threshold. As a result, the set of geodesic distances could guide the histopathologist in the correct measurement of Breslow thickness, after the identification of the deepest melanocyte.

By evaluating the overall spatial distribution of cells, we observed a significant correlation between the distances annotated by Operator 2 and the 80th percentile of the cell distribution (y = 0.88x, R^2^ of 0.92, Pearson correlation of 0.96, *p*-value ≤ 0.001). Examples of the obtained distributions are shown in Figure 4.

## 4. Discussion

Over the years, numerous studies have confirmed that tumor thickness is the single most important predictor of survival from melanoma [8,9]. However, the correlation between tumor thickness and prognosis is not absolute. In fact, a small number of patients with so-called “thin” melanomas (defined as a lesion of a thickness less than 0.8 mm in the latest AJCC staging) still develop metastases, while some patients with thicker melanomas do not [51,52,53].

Performing Breslow measurements in clinical practice comes with several challenges. Despite their expertise, using a microscope, pathologists can examine only a small portion of the tumor at a time, potentially missing the broader context and overall cellular distribution. Furthermore, the presence of dirties, bubbles, and epidermal ulcerations poses irreversible issues in some real cases: these kinds of issues cannot certainly be solved by any human or AI-based solution, since they represent intrinsic errors within the acquisition procedures.

Moreover, as is the case with any distance measurement, uncertainty arises from the definition of the starting and ending points of the Breslow depth, as well as the orientation of the instrument used. In addition, the epidermal layer is typically curved and contains multiple invaginations, and this hinders the repeatability of the measurement. Tissue fixation adds to the complexity of the measurement, since it often causes further distortion and shrinkage, compromising the accuracy of the micrometer alignment [24].

The heterogeneity of tissue slices collected from the same patient can also result in significant differences in the measured tumor thickness. Additionally, distinguishing in situ melanoma from superficially invasive melanoma can be particularly challenging. This also applies to differentiating invasive melanoma with a nevoid appearance from melanoma associated with a nevus [54,55,56].

Our findings demonstrate that variability in the distances recorded by different operators stems from the subjective nature of the measurement, which entails identifying a single point, namely the deepest melanocyte, within a complex tissue structure. The strong linear relationship between angular dispersion and Breslow thickness dispersion (R^2^ = 0.67, *p* ≤ 0.001) confirms that increasing difficulty in identifying the correct perpendicular direction directly propagates into increasing measurement uncertainty. Consistently, five out of seven operators showed a statistically significant overestimation relative to the mathematically computed geodesic distance, indicating that visually estimating the shortest path to a curved epidermal surface is systematically prone to upward bias [24]. Statistically, any measurement based on a single outlier is prone to high variability. Thus, more robust statistical indicators should be considered. To this end, an objective comparison between Breslow thickness and the spatial distribution of cells can be made by evaluating the distribution of distances between all cells and the granular layer surface.

Furthermore, the possibility of automatically identifying the number and spatial distribution of cells within a tissue sample could speed up clinical evaluation. Moreover, the semantic segmentation of the tissue components, along with their morphological characterization and localization in terms of pixels of the digitized sample, has the potential to minimize the sources of error, introducing a level of objectivity that is still lacking in conventional pathological assessments.

It is interesting to note that, in his original work, Breslow identified melanoma depth as the histological feature having the most robust and reliable prognostic value, despite the lack of evidence that Breslow thickness directly reflects tumor burden [25]. This suggests that tumor thickness may serve as a quantitative marker for the biological processes that drive melanoma progression and spreading.

Indeed, several studies have attempted to associate patterns in gene expression and miRNA dysregulation with Breslow thickness [2,18,57]. Proteins whose expression correlates with tumor thickness are often involved in cell adhesion, survival, and invasiveness [2,18,58]. Notably, E-cadherin, a key adhesion molecule between keratinocytes and melanoma cells, is frequently downregulated at an early stage in melanoma, promoting an increase in tumor motility and invasiveness [59,60].

From this perspective, Breslow thickness should not be interpreted as a measure of tumor mass, but rather as an indicator of the motility, invasion, and progression of the melanoma cells. Therefore, the density and spatial distribution of cells within the tissue slice are key factors that should be considered to better contextualize the Breslow measurement [61,62].

The importance of cell distribution is underscored by the reasonable assumption that two melanomas with the same Breslow thickness, but different cell densities at the measurement point, may be characterized by a different degree of invasiveness and prognostic trends [59,60]. As a matter of fact, the tumor with fewer invasive cells is likely to have a more favorable prognosis.

To address this, the term “Breslow density” was introduced in 2017 to describe the estimated melanoma cell area at the position where Breslow thickness is measured [25]. The aim is to demonstrate that Breslow density could be a simple and reproducible morphological biomarker of prognostic relevance. Indeed, preliminary studies have shown that combining Breslow density with Breslow thickness improves the 5-year survival prediction [25].

These findings highlight the value of correlating Breslow thickness with both cell distribution and density within tissue samples. Using our semi-automated software, it was possible to obtain the spatial distribution of cells in all tissue samples. We suggest that Breslow thickness could be associated with the distance from the granular layer surface, providing a more robust statistical metric that bypasses the selection of a specific cell. This result would emphasize the advantages of integrating AI into digital pathology, making it possible to refine standard histopathological measures and identify more robust statistical indicators that could help minimize human subjectivity. Several limitations of the present study should be acknowledged. First, the starting point of the measurement was fixed to the deepest melanocyte identified by Operator 2, deliberately isolating the directional component of variability; the contribution of disagreement in identifying the deepest cell itself was therefore not assessed and should be investigated in future unconstrained studies. Second, the dataset was limited to 40 single-institution pT1a samples, restricting generalizability to thicker or more heterogeneous tumors. Third, the observed correlation between the 80th percentile of the cell distance distribution and operator-annotated Breslow values (R^2^ = 0.92) is a preliminary and exploratory finding: it could work as a statistical proxy and suggests that percentile-based summaries may offer greater robustness than a pure maximum against outlier cells, but this requires dedicated validation on independent datasets before any clinical interpretation can be considered. Finally, full automation of the pipeline, including validated melanocytic cell classification, was not achieved in this study and remains a direction for future development.

## Data Availability

The WSI raw data that support the findings of this study are available from the corresponding author upon reasonable request. The code developed for the automated segmentation of histological cells is part of the SYNTHEMA EU project, and it will be released on the GitHub page dedicated to the project, available at https://github.com/synthema-project, accessed on 1 May 2026. The entire dataset, including measurements collected by operators and those automatically generated by the proposed pipeline, is available as Supplementary Data for the manuscript.

## Supplementary Materials

The following supporting information can be downloaded at: https://www.mdpi.com/article/10.3390/e28060643/s1, Dataset: Breslow_dataset.csv.
